# Diagnostik, Prävention und Therapie des Lymphödems

**DOI:** 10.1007/s00508-020-01766-y

**Published:** 2020-11-23

**Authors:** Tonatiuh Flores, Konstantin D. Bergmeister, Anton Staudenherz, Karin Pieber, Klaus F. Schrögendorfer

**Affiliations:** 1 Karl Landsteiner Privatuniversität für Gesundheitswissenschaften, Dr. Karl-Dorrek-Straße 30, 3500 Krems, Österreich; 2 Klinische Abteilung für Plastische, Ästhetische und Rekonstruktive Chirurgie, Universitätsklinikum St. Pölten, Dunant-Platz 1, 3100 St. Pölten, Österreich; 3 Klinisches Institut für Nuklearmedizin, molekulare Bildgebung und spezielle Endokrinologie, Universitätsklinikum St. Pölten, Dunant-Platz 1, 3100 St. Pölten, Österreich; 4 Klinisches Institut für Physikalische Medizin und Rehabilitation, Universitätsklinikum St. Pölten, Dunant-Platz 1, 3100 St. Pölten, Österreich

## Definition

Das Lymphsystem bildet sich bereits in der fünften embryonalen Entwicklungswoche

Das Lymphsystem besteht aus lymphatischen Organen und dem Lymphgefäßsystem und stellt als Abwehrsystem gegenüber Krankheitserregern, einen Teil des Immunsystems dar. Zusätzlich dient es dem Transport von Flüssigkeit und Lipiden. Es entsteht bereits in der fünften Entwicklungswoche und schließt sich, ausgehend von Lymphkapillaren, früh dem venösen System an. Das Lymphgefäßsystem verläuft parallel zu den Arterien und Venen des Körpers. Es ist von Lymphknoten zwischengeschalten, bis es schlussendlich als Ductus thoracicus in den linken, beziehungsweise als Ductus lymphaticus dexter, in den rechten Venenwinkel mündet.

Liegen morphologische und funktionelle Störungen des Lymphsystems vor, spricht man von einem Lymphödem

Das Ödem ist primär ein klinisches Symptom und beschreibt den Austritt von Flüssigkeit aus dem Gefäßsystem in den interstitiellen Raum. Hierbei müssen per Definition morphologische und funktionelle Störungen im Lymphsystem vorliegen um als Lymphödem bezeichnet zu werden. Kann die im interstitiellen Raum befindliche Flüssigkeit nicht mehr ausreichend abtransportiert werden, so ist die Homöostase zwischen Bildung und Abtransport gestört. Es folgt ein Rückstau von Flüssigkeit und konsekutiv entsteht ein Ödem. Wegen des hohen Anteils an Proteinen, resultiert in weiterer Folge ein Anstieg des kolloid-osmotischen Drucks, wodurch vermehrt Flüssigkeit in den interstitiellen Raum gezogen wird. Bei längerem Fortbestehen führt dieses Lymphödem zu chronischen Schwellungen und Entzündungsreaktionen, der Ablagerung von Fettzellen und letztendlich zu fibrotischen, irreversiblen Umbauprozessen.

## Klassifikation

Das Lymphödem kann in ein primäres und ein sekundäres Lymphödem unterteilt werden

Ursachen des Lymphödems können einerseits angeboren sein, andererseits durch chronischen Entzündungen, nach Verletzungen oder nach Krebstherapien entstehen. Auch wenn beiden Formen des Lymphödems ähnliche Pathomechanismen zugrunde liegen, so unterscheiden sie sich stark im Verlauf und sprechen unterschiedlich auf entsprechende Therapien an [1].

### Primäres Lymphödem

Das primäre Lymphödem hat eine Häufigkeit von 1:100.000

Das primäre Lymphödem stellt eine sehr seltene Krankheitsform dar. Meist kommt es hierbei, bedingt durch Entwicklungsstörungen, zu einer Unterentwicklung, einem partiellen Fehlen, oder einer fehlerhaften Ausbildung von Lymphgefäßen. Ein komplettes Fehlen von Lymphgefäßen ist nicht mit dem Leben vereinbar. Es können sowohl die oberen als auch die unteren Extremitäten als Ganzes betroffen sein, wobei die Beine häufiger betroffen sind. Primärformen können weiter unterteilt werden je nach: Vererbungsform (sporadisch oder genbezogen), der Lokalisation (viszeral oder systemisch), oder Zeitpunkt des Auftretens (angeboren, vor der Pubertät, oder im Erwachsenenalter). Die häufigste Form ist angeboren und entsteht im Laufe der Pubertät [1].

Beispiele für angeborene Formen des Lymphödems sind die Meige-Krankheit, welche in der Pubertät auftritt, die Milroy-Krankheit, welche sich kurz nach der Geburt manifestiert und das Nonne-Milroy-Meige-Syndrom, welches mit Minderwuchs und mentaler Retardierung einhergeht.

### Sekundäres Lymphödem

Die häufigste Ursache für sekundäre Lymphödeme in Industriestaaten sind Krebstherapien, insbesondere nach Brustkrebs

Das sekundäre Lymphödem ist die häufigste Form des Lymphödems. Es tritt als Ergebnis direkten oder indirekten Schadens auf. Die häufigste Ursache stellt die lymphatische Filariose, eine in Entwicklungsländern häufige Krankheit durch parasitären Befall, mit einer Prävalenz von 140 bis 250 Mio. Betroffene, dar. So genannte Filarien vermehren sich hierbei in befallenen Lymphknoten, wodurch der Abtransport der anfallenden Lymphe vermindert wird. Diese Form kommt in Industriestaaten äußerst selten vor [1].

**Abb. 1:**
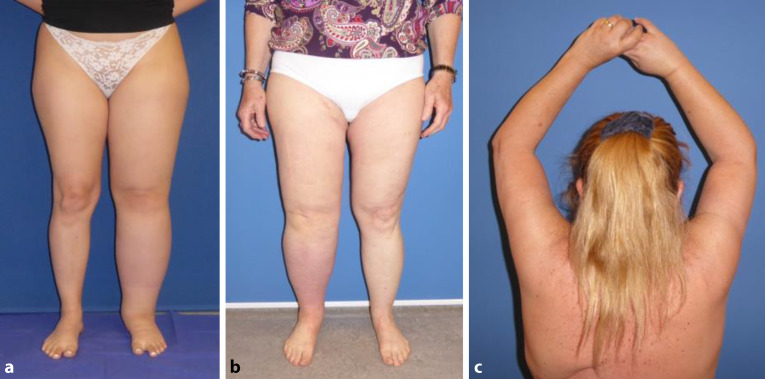
**a** Idiopathisches Lymphödem der linken unteren Extremität bei einer 19-jährigen Frau. **b** Lymphödem nach Uteruskarzinom mit Lymphknotendissektion der rechten unteren Extremität bei einer 62-jährigen Frau C: Lymphödem nach Mastektomie und Axilladissektion der rechten oberen Extremität bei einer 58-jährigen Frau

Weitaus häufigere Ursachen für die Manifestation des sekundären Lymphödems in Industrieländern sind Infektionen, Traumata, die chronisch venöse Insuffizienz, Übergewicht oder iatrogene Ursachen, insbesondere nach Krebstherapien. Die Entfernung, oder Bestrahlung der Lymphabflusswege, aber auch Chemotherapien in der Therapie des Mammakarzinoms, sind dabei die häufigste Ursache für sekundäre Lymphödeme (Abb. 1) [2, 3].

## Epidemiologie

Frauen sind häufiger betroffen als Männer

Die genaue Zahl primärer und sekundärer Lymphödeme ist unklar, da sich die Häufigkeiten weltweit stark unterscheiden. Das primäre Lymphödem kommt mit einer Häufigkeit von 1:100.000 vor und ist häufiger präsent in Entwicklungsländern [4]. Frauen sind ca. 1:4,5 × bis 1:6,1 × öfter betroffen als Männer [5]. Ebenso ist die Entwicklung des Lymphödems an den unteren Extremitäten statistisch häufiger. In ca. 30 % der primären Lymphödemen liegt eine genetische Erkrankung vor (meist betroffen ist das VEGF-Gen) [1].

Die Inzidenz des sekundären Lymphödems nach Brustkrebs liegt bei ca. 19,9 %

Die Gesamtinzidenz des sekundären Lymphödems wird auf ca. 0,13–2 % geschätzt [6]. Die Entfernung von Lymphknoten, im Zuge einer Krebstherapie, aber auch die Bestrahlung solcher, sind die Hauptursache sekundärer Lymphödeme in Industrieländern. Mit einer Inzidenz von ca. 5500 Neuerkrankungen pro Jahr, tritt dies am häufigsten beim Mammakarzinom auf [7]. Obwohl sich die Radikalität der Tumorchirurgie in den letzten Jahren bei gleichbleibender onkologischer Sicherheit reduzierte, liegt die Inzidenz sekundärer Lymphödeme innerhalb von 12–24 Monaten nach axillärer Lymphknotenentfernung bei Mammakarzinom noch immer bei ca. 19,9 %. Aber auch kleinere Eingriffe in das Lymphsystem, wie zum Beispiel die Entfernung des Wächterlymphknotens, führen in 5–7 % der Fälle zu sekundären Lymphödemen [8, 9, 10]. Bei gynäkologischen Tumoreingriffen liegt die Lymphödemrate bei ca. 20 % [11].

## Staging

Das Lymphödem kann anhand der Klassifikation der International Society of Lymphology in vier Stadien eingeteilt werden.

Die Stadien des Lymphödems können nach verschiedenen Klassifikationen eingeteilt werden (u. a. American classification, Italian classification, Brazilian classification). Die geläufigste Einteilung ist die der International Society of Lymphology (Stadien 0–3) [1].

### Stadium 0

Im Stadium 0 des Lymphödems liegt noch keine Schwellung vor

Das Intervallstadium, oder auch latentes Stadium, beschreibt einen subklinischen Verlauf. Das Lymphsystem ist hierbei zwar geschädigt, allerdings ist der Abtransport von Flüssigkeit noch regelrecht. Es tritt somit keine Schwellung auf. Symptome dieser Patienten beschränken sich meist auf ein Schweregefühl, Unwohlsein, oder frühes Ermüden der betroffenen Extremität. Lymphszintigraphien können in diesem Stadium bereits einen pathologischen Verhalt detektieren.

### Stadium I

Ein Hochlagern der betroffenen Extremität, kann zu einer Verbesserung der Symptomatik im Stadium I führen

In diesem Stadium ist eine Schwellung bereits manifest, jedoch reversibel. Hier treten noch keine Gewebsveränderung auf. Das Ödem ist weich und auf Druck verbleibt eine sichtbare Delle. Patienten in diesem Stadium profitieren sehr von einem konservativen Schema. Ein Hochlagern der betroffenen Extremität, oder das Bandagieren, bieten meist zu Beginn eine ausreichend suffiziente Therapie.

### Stadium II

Das Lymphödem ist in diesem Stadium durch Hochlagern nicht mehr reversibel. Auf Druck verbleibt hier keine Delle mehr, da bereits ein fibrotischer Umbau des Unterhautgewebes begonnen hat. Die Konsistenz des Ödems ist je nach Fibrosegrad, bereits verhärtet. Ein konservatives Therapieschema ist in diesem Stadium noch anwendbar. Bei konservativer Therapieresistenz ist hierbei bereits eine chirurgische Intervention indiziert.

### Stadium III

Die Elephantiasis ist eine krankhafte Vergrößerung der Extremitäten durch Lymphstau

Dieses Stadium beschreibt bereits den Zustand der Elephantiasis. Die betroffene Extremität ist stark geschwollen und betroffene Patienten in der Bewegung stark eingeschränkt. Fibrotische und adipöse Ablagerungen können zu einer gestörten Blutversorgung der Haut führen. Akanthosen, Hyperkeratosen, aber auch Ulzerationen können auftreten.

## Diagnostik

Die Grundlage der Diagnostik bilden die Anamnese, die Inspektion und die Palpation.

Der wichtigsten Bestandteile der Diagnostik sind Anamnese, Inspektion und Palpation

Durch die in der Basisdiagnostik erhobenen Befunde, kann, sofern keinerlei Komorbiditäten vorliegen und sich das Lymphödem im Stadium I oder höher befindet, die Diagnose gestellt werden. Bei Komorbiditäten (insbesondere Krebserkrankungen), dem Vorliegen eines Frühstadiums, Planung einer chirurgischen Intervention, oder initial unschlüssiger Befundung, ist jedoch eine bildgebende Diagnostik zusätzlich notwendig.

### Anamnese

Die Anamnese gliedert sich in eine Allgemeinanamnese und eine Ödemanamnese. Im Zuge der Allgemeinanamnese sollte auch die Familienanamnese, insbesondere familiäre Erkrankungen wie Diabetes mellitus, chronisch venöse Insuffizienz, oder gehäufte Lymphödeme, erhoben werden. Zusätzlich sollte man Voroperationen, Infektionen, sowie Traumata abklären und auch die Medikamenteneinnahme abfragen. Sollte in der Patientenanamnese eine Krebserkrankung vorkommen, werden hierfür dazugehörige Vorbefunde zur genauen Abklärung benötigt, ob ein Zusammenhang zwischen Lymphödem und Krebserkrankung vorliegt.

Ein essenzieller Aspekt der Anamnese ist die Frage nach Krebserkrankungen

Die gezielte Ödemanamnese beinhaltet vor allem den Beginn und zeitlichen Verlauf der Krankheit, sowie wie die Lokalisation. Weitere Punkte sind Begleiterkrankungen wie Schmerzen, Schwellungen, Hauterscheinungen wie Erysipele, oder erhöhte Hämatomneigung. Eventuelle Vermeidungs- und Verbesserungsstrategien (wie das Erhöhen der betroffenen Extremität im Alltag) der Patienten sowie das Erfragen von Kurzatmigkeit und Alltagsaktivitäten sollen ebenso Teil der Anamnese sein.

### Inspektion

Die Inspektion soll sowohl am stehenden als auch am liegenden Patienten durchgeführt werden

Im Zuge der Inspektion erfolgt die Stadieneinteilung des Patienten. Der Patient soll die betroffene Region entkleiden und sowohl im Stehen als auch im Liegen beurteilt werden. Besonderes Augenmerk liegt auf der Lokalisation, der Schwellung und der Umfangsdifferenz bezogen auf die gesunde Extremität (sofern das Lymphödem einseitig vorliegt). Liegt das Lymphödem beidseitig vor, so ist der Umfang beider Extremitäten als eigenständiger Wert anzugeben.

### Palpation

Bei der Palpation, werden sowohl die betroffenen Extremitäten als auch inguinale, sowie axilläre Lymphknoten untersucht. Lymphknoten sollen auf Größe, Konsistenz, Druckdolenz und Verschieblichkeit beurteilt werden. Die Beschaffenheit des Ödems (weich, teigig, prall, fibrotisch, hart), sowie das Verbleiben einer Delle auf Druck werden ebenfalls untersucht.

Das Stemmer’sche Zeichen dient als diagnostisches Hilfsmittel. Hierzu wird die Haut an der proximalen Phalanx der zweiten Zehe angehoben. Ist dies nicht möglich, so ist das Stemmer’sche Zeichen positiv und weist auf ein Lymphödem hin. Ist das Stemmer’sche Zeichen negativ, kann jedoch nicht ausgeschlossen werden, dass kein Lymphödem vorhanden ist. Ergänzend zur Palpation werden die aktive und passive Gelenksbeweglichkeit, die Funktion (Faustschluss, Zangengriff), die Sensibilität, die Narbenqualität, das Nagel- und Haarwachstum, sowie die Hauttemperatur beurteilt. Zur Berechnung der Volumendifferenz dient die Formel nach Kuhnke. Sie beschreibt die Berechnung eines nach oben hin offenen Zylinders.

### Bildgebende Diagnostik

Die Volumenmessung nach Kuhnke bietet eine exakte Bemessung des Volumens bei Lymphödemen

Eine bildgebende Diagnostik ist vor allem notwendig, wenn ein Lymphödem klinisch nicht eindeutig verifiziert werden kann. Hiermit kann die Diagnose gesichert und ggf. die zugrunde liegende Ursache des Lymphödems identifiziert sowie Verlaufskontrollen durchgeführt werden.

Kann am Fußrücken keine Hautfalte gebildete werden, ist das Stemmer’sche Zeichen positiv

Die gängigen diagnostischen Verfahren sind die Lymphszintigraphie und die Fluoreszenzlymphangiographie (Indocyaningrün). Bei diesen Untersuchungen kann bereits im Frühstadium ein verlangsamter Lymphabfluss nachgewiesen werden. Die Magnetresonanz, oder die Computertomographie, können ein Lymphödem erst ab Stadium II erfassen. Die Ultraschalluntersuchung dient insbesondere der präoperativen Planung.

#### Lymphszintigraphie

Die Lymphszintigraphie ist eine funktionelle bildgebende Diagnostik und stellt den Gold-Standard der Lymphödemdiagnostik dar.

Die bildgebende Diagnostik dient unter anderen zur Operationsplanung

Im Zuge dieser Untersuchung wird ein radioaktiver Tracer (meist Technetium, Tc^99^) subkutan in der Peripherie der zu untersuchenden Extremität appliziert (ca. 20 bis max. 60 MBq pro Applikationsstelle). Dieser Marker wird innerhalb einer bestimmten Zeiteinheit ausschließlich vom Lymphsystem abtransportiert. Typische Injektionsstellen sind hierbei der erste oder zweite Zwischenzehenraum für die untere Extremität, bzw. der zweite oder dritte Zwischenfingerraum für die obere Extremität. Dadurch kann der Abfluss über das Lymphsystem abgebildet werden.

MRT und CT können ein Lymphödem erst ab Stadium II erfassen

Die Applikation erfolgt zeitgleich in beide Extremitäten; anschließend werden Aufnahmen bis zu 2 h nach Injektion angefertigt. Zur Beschleunigung des Lymphflusses, wird eine Mobilisation durchgeführt (z. B. Treppensteigen oder Faustschluss), Dadurch ermöglicht die Lymphszintigraphie eine morphologische Darstellung der oberflächlichen oder tiefen Lymphgefäße und auch der Lymphknoten.

Die Darstellung der Lymphgefäße erfolgt simultan in zwei Ebenen (anterior und posterior). Durch Anwendung der SPECT-Szintigraphie (Single Photon Emission Computed Tomography) können auch dreidimensionale Abbildungen erstellt werden. Eine weitere Darstellungsmöglichkeit, ist die Kombination einer SPECT-Aufnahme mit einem CT (Computertomographie). Dies erlaubt eine hybrid Darstellung der Lymphgefäße in ihrer anatomischen Umgebung.

Die Lymphszintigraphie dient der funktionellen Darstellung der Lymphödeme

Diese Untersuchung ist vor allem zur präoperativen Beurteilung des Lymphabflusssystems notwendig und dient der Bestimmung, bzw. Darstellung von Verletzungen oder Klappeninsuffizienz von Lymphgefäßen oder auch proximalen Lymphabflussbehinderungen/-störungen.

#### Indocyaningrün Fluoreszenzlymphangiographie

Die Lymphszintigraphie kann mit SPECT und CT erweitert werden und so den Bezug von Lymphgefäßen zu umliegenden Strukturen darstellen

ICG dient zur Echtzeitüberprüfung des Lymphabflusses

**Abb. 2:**
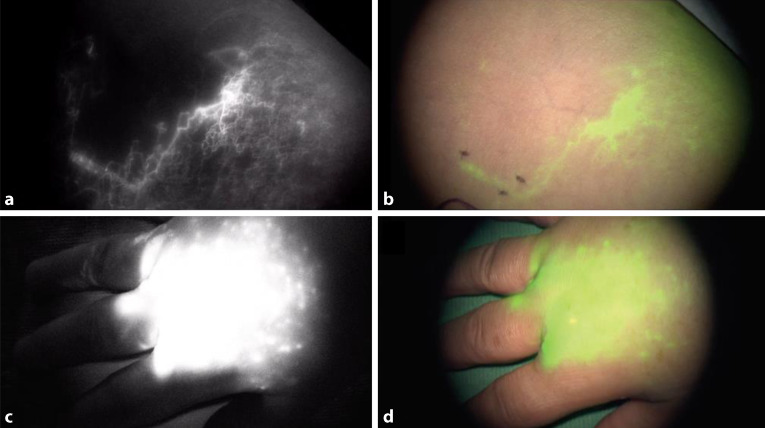
Lymphgefäßdarstellung des Unterarms via ICG Angiographie: **a** Infrarotaufnahme (ICG) eines normalen Lymphgefäßes (in *weiß* dargestellt), **b** Nativaufnahme des selben Gefäßes mit ICG-Überlagerung (in *grün* dargestellt), **c** Infrarotaufnahme (ICG) eines dermalen Backflows bei insuffizientem lymphatischen Abstrom und Rückstau über die Haut in Stadium II des Lymphödems (*weiße Wolke*), **d** Nativaufnahme des selben dermalen Backflows mit ICG-Überlagerung (*grüne Wolke*)

Indocyaningrün (ICG) ist ein autofluoreszierender Stoff. Es dient der Echtzeiterfassung von ausschließlich oberflächlichen Lymphgefäßen. Die Applikationsstellen von ICG sind gleich zur Lymphszintigraphie. Nach ausreichender Wartezeit können mittels Infrarotkamera, die Lymphgefäße dargestellt werden (Abb. 2). Ist der Lymphabfluss gestört erfolgt die Verteilung von ICG als diffuser „Backflow“ über die Haut.

Die Lymphszintigraphie kann ein Lymphödem bereits im Stadium 0 erfassen

Außerdem kann via der ICG-Angiographie intraoperativ die Durchgängigkeit von lymphovenösen Anastomosen (LVA) überprüft werden. Der Chirurg ist dadurch in der Lage mögliche Leckagen oder Gefäßverschlüsse nach Vernähen einer Anastomose (und erneuter Applikation von ICG), festzustellen und zu korrigieren. Mithilfe von ICG kann außerdem für die präoperative Planung ein Lymphknoten-Mapping erstellt werden um die Entnahme von freien Lymphknotenlappenplastiken (VLNT, vascularized lymph node transfer) zu planen [12].

Die Darstellung der Lymphgefäße mittels ICG und Infrarotkamera ist somit eine einfache und kostengünstige Untersuchungsmethode, welche ohne Strahlenbelastung einhergeht und ambulant durchgeführt wird.

#### Ultraschall

Die Sonographie dient vor allem der Verlaufskontrolle des Lymphödems

Die Sonographie dient der Verlaufskontrolle von Lymphödemen. Mit diesem können interstitielle Flüssigkeit und der Fibrosegrad, sowie die Akkumulation von Fettzellen in der Subkutis abgebildet werden. Diese Veränderungen können mittels Ultraschall frühestens ab Stadium I nachgewiesen werden und stellen kein pathognomisches Kriterium für ein Lymphödem dar. Zur Detektion eines Lymphödems selbst ist der Ultraschall daher nicht geeignet.

Die Ultraschalluntersuchung stellt allerdings eine verlässliche Methodik zur Detektion, oder dem Ausschluss differenzialdiagnostischer Krankheitsbilder wie der chronisch venösen Insuffizienz (CVI) oder dem Lipödem dar. Jedoch schließt die Bestätigung einer CVI nicht aus, dass auch ein Lymphödem simultan vorliegen kann.

Die präoperative Planung für Lymphknotentransfers kann mit Ultraschall durchgeführt werden

Für die präoperative Planung dient die Sonographie zusätzlich zur Größenbestimmung und Lokalisation der zu entnehmenden Lymphknoten, um so den freien Lymphknotentransfer zu vereinfachen.

## Therapie

Prinzipiell sollte bei Patienten mit hohem Risiko für ein Lymphödem immer eine Primärprävention etabliert werden, um das Entstehen zu verhindern. Ebenso soll das Fortschreiten des Lymphödems verhindert werden. Bei einem manifestem Ödem erfolgt die Basistherapie des Lymphödems immer konservativ und wird bei Bedarf um chirurgische Maßnahmen erweitert. Die Art und die Möglichkeit der Therapie richtet sich nach Stadium des Befundes, sowie nach der Komorbidität der Patienten (insbesondere Krebserkrankungen). Essentiell für die Therapieentscheidungen bei sekundären iatrogenen Lymphödemen ist die initial durchgeführte Therapiemaßnahme (z. B. Lymphknotendissektion der Axilla und Bestrahlung). Durch die Möglichkeiten der modernen plastischen Chirurgie können heute schonende Verfahren mit kurzem Krankenhausaufenthalt angeboten werden, die meist rasche Besserung innerhalb weniger Tage bis Wochen bieten.

### Primärprävention

Die Prävention von sekundären Lymphödemen spielt eine entscheidende Rolle bei Patienten mit hohem Risiko, wie vor allem bei Brustkrebspatientinnen. Ursächlich hierfür ist vor allem die Dissektion oder Resektion von drainierenden Lymphknoten sowie postoperative Strahlen- und Chemotherapie.

Die Therapie des Lymphödems soll patientenorientiert erfolgen

Für die Prävention von Lymphödemen gibt es derzeit nur wenig evidenzbasierte Literatur. Viele der Angaben beruhen auf Expertenkonsensus und empfehlen Hautpflege, körperlicher Aktivität, sowie die Verwendung von Kompressionswäsche [13].

### Konservative Therapie

Die konservative Therapie des Lymphödems zielt auf eine Reduktion der interstitiellen Flüssigkeit ab, wodurch der Lymphabfluss verbessert und eine Progression in höhere Stadien verhindert werden soll. Hierfür wurde die dekongestive oder komplexe physikalische Entstauungstherapie (KPE) entwickelt, welche sich aus mehreren unterschiedlichen Therapien zusammensetzen. Diese beinhaltet die manuelle Lymphdrainage, die Kompressionstherapie (Entstauungsphase, Phase I und Erhaltungsphase, Phase II), sowie gezielte körperliche Übungen, Hautpflege und falls erforderlich Hautsanierung, sowie Aufklärung und Schulung zur Selbsttherapie [14].

#### Manuelle Lymphdrainage

Die KPE ist ein multimodales Therapiekonzept, welches mit chirurgischen Therapien kombiniert werden kann

Durch die manuelle Lymphdrainage (MLD) soll das Lymphsystem zum vermehrtem Abtransport von Zwischenzellflüssigkeit angeregt werden. Dies geschieht durch das Prinzip der Dehnung der Haut und Unterhaut durch vier Grundgriffe (stehender Kreis, Pumpgriff, Schöpfgriff und Drehgriff nach Emil Vodder), welche das oberflächliche Lymphsystem anregen.

Diese Grundgriffe werden vor allem im Stadium 0 und 1 des Lymphödems eingesetzt. Zusätzlich wird durch den manuellen Druck der Sympathikotonus herabgesetzt, welcher sonst eine erhöhte Konstriktion von Lymphgefäßen verursacht. Des Weiteren werden durch die Grundgriffe ein gerichteter Druck von distal nach proximal erzeugt, welcher den Flüssigkeitstransport in den Lymphbahnen erhöht.

In den Stadien II und III, bzw. bei fibrotisch verändertem subkutanem Gewebe, müssen der manuelle Druck entsprechend adaptiert und weitere Drainagetechniken appliziert werden. Hierbei kommen die Ödem- und Fibrosetechniken zum Einsatz. Die Ödemtechnik hat zum Ziel proteinreiches Ödem zusätzlich über die tiefer gelegenen Lymphgefäße zu drainieren. Bei der Fibrosetechnik gilt es lymphostatische Fibrosen aufzulösen. Diese Art der Lymphdrainage kommt erst dann zur Anwendung, wenn die betroffene Extremität durch die vier Grundgriffe bereits entstaut wurde. Anschließend werden Kompressionsbandagen angelegt, um die Entstauung zu erhalten. Liegen ein schmerzhaftes Lipödem, Tiefvenenthrombose, Varizen, oder Schmerzsymptomatik anderer Genesen vor, bzw. sind Patienten antikoaguliert, oder leiden an Hämophilie, dürfen diese Drainagearten nicht angewendet werden. Allgemeine Kontraindikationen beinhalten die dekompensierte Herzinsuffizienz, pAVK Stadium III/IV, Erysipele oder erosive Dermatosen [15].

Im klinischen Alltag können ebenso die apparative Lymphdrainage und die Anwendung von Lymphtapes, symptomlindernde Therapieansätze darstellen.

#### Kompressionstherapie

Die manuelle Lymphdrainage erhöht den lymphatischen Rückfluss

Die konservative Therapie des Lymphödems ist auf zusätzliche Kompression von außen angewiesen, da die Lymphgefäße beim Lymphödem bereits in ihrer Funktion und Struktur geschädigt sind. Weiters wird durch die Kompressionstherapie der Entstauungseffekt der MLD beibehalten und somit eine Re-Akkumulation verhindert. Gleichzeitig wird der venöse Druck reduziert, hierdurch die Ultrafiltration der Blutgefäße reduziert und damit der Lymphfluss in den noch funktionierenden Lymphgefäßen erhöht.

Bei höheren Stadien des Lymphödems muss die MLD um die Fibrosetechnik und die Ödemtechnik erweitert werden

Kurzzugbandagen stellen die erste Wahl der Kompressionstherapie dar. Mit einer Dehnbarkeit von bis zu 60 %, halten sie einen ausreichend hohen Arbeitsdruck während muskulärer Tätigkeit aufrecht. Der dadurch aufgebaute Druck führt dem Lymphsystem bei Arbeitsphasen ausreichend Flüssigkeit zu, um ödematöser Akkumulation entgegenzuwirken. Gleichzeit führen Kurzzugbandagen zu einem niedrigen Ruhedruck, wodurch oberflächliche Venen und Lymphgefäße in Ruhephasen, nicht komprimiert werden und somit einen adäquaten Rücktransport gewährleisten. Langzugbandagen, mit einer Elastizität von bis zu 140 % führen während der oben genannten Phasen, zu einem gegenteiligen Effekt [15]. Zusammen mit der manuellen Lymphdrainage zählen Kompressionsbandagen zur Phase I (Entstauungsphase) der KPE.

Die Kompressionstherapie dient dem Entgegenwirken der Re-Akkumulation interstitieller Flüssigkeit

Sobald die betroffene Extremität erfolgreich entstaut wurde, erfolgt die Phase II (Erhaltungsphase) der Kompressionstherapie mittels Kompressionskleidung. Diese Form der Kompression bildet von distal nach proximal einen Druckgradienten. Je nach Druck erfolgt die Einteilung in drei (bei Rundstrick), bzw. vier Kompressionslevel (bei Flachstrick). Das Anlegen mehrerer Kompressionsstücke übereinander verdoppelt den entsprechenden Druck allerdings nicht. Zusätzlich können Kompressionsbandagen an Körperstellen, distal der Kompressionskleidung, angelegt werden.

Kurzzugbandagen unterstützen das Lymphsystem während Arbeits- und Ruhephasen

Abhängig von der Produktionsart (Flachstrick, Rundstrick) variieren die verschiedenen Drucklevel. Während Rundstrick zwischen 20–50 mm Hg Druck aufbauen kann, bietet Flachstrick ein Spektrum von 18- über 50 mm Hg. Flachstrick-Kompressionswäsche kann zusätzlich nach Maß angefertigt werden, was zu einem besseren Anliegen an der entsprechenden Extremität führt. Dies ist vor allem bei höheren Stadien, bzw. bei höherem

Flachstrick-Kompressionswäsche nach Maß hat den Vorteil der optimaleren Kompressionsverteilung

Schweregrad vorteilhaft. Für einen erwünschten Effekt der Kompressionswäsche muss diese permanent getragen werden. Die Wahl der Kompressionsklasse, sowie die Art der Herstellung und die Form, müssen je nach Patient, individuell angepasst werden. Je nach Lokalisation gibt es Kompressionshandschuhe, -Ärmel, oder -Strümpfe oder Pelotten (geeignet für fibrotische Stellen).

#### Körperliche Übungen

Adäquate sportliche Betätigung zählt zur Phase I der KPE und soll in jedem Fall in Phase II weitergeführt werden. Muskel und Gelenkspumpen werden aktiviert, wodurch ein erhöhter Rückfluss über die Lymphgefäße verursacht wird. Lymphödemorientierter Sport in Kombination mit Kompressionstherapie erhöht diesen Effekt und sollte unbedingt unter Kompression durchgeführt werden.

Das Zwerchfell und dessen Aufwärts- und Abwärtsbewegung führen zu einem erhöhten Lymphrückfluss über abdominale Lymphbahnen. Sowohl die Bauch-, als auch die Brustatmung erzielen den gewünschten Effekt. Es empfiehlt sich mit Atemübungen anzufangen und diese in die weiterführende sportliche Betätigung miteinfließen zu lassen.

Ausdauersport wie beispielsweise Radfahren, Schwimmen, oder Nordic Walking, haben einen positiven Effekt auf die körperliche Leistung und führen zu einem verbesserten Lymphfluss.

### Chirurgische Therapien

Die chirurgische Therapie erfolgt nach konservativem Therapieversuch und ist immer an die begleitende konservative Therapie gebunden. Die Art der Operation richtet sich hierbei immer nach dem Verlauf, dem Stadium und der Lokalisation des Lymphödems. Generell zeigen sekundäre Lymphödeme ein besseres Ansprechen auf chirurgische Verfahren, als primäre Erscheinungsformen.

Noch bevor sich ein Lymphödem etabliert, empfiehlt sich ein präventives Vorgehen bei onkologischen Ersteingriffen, wie das Schonen von Lymphgefäßen.

Körperliche Übungen erzielen den besten Effekt, wenn sie mit Kompressionswäsche kombiniert werden

Bei den plastisch chirurgischen Verfahren zur Behandlung des Lymphödems werden zwei Gruppen unterschieden:

1. Reduktive Verfahren, dazu zählen direkte Exzision und Liposuktion und
2. Physiologisch-rekonstruktive Verfahren, dazu zählen Lymphbypass mit Lymphgefäßtransfer, lymphovenöse Anastomosen (LVA) und die freie Transplantation von Lymphknoten (vascularized lymh node transfer, VLNT).

Prinzipiell wird vom Seniorautor immer eine physiologische Rekonstruktion empfohlen und angestrebt. Die Liposuktion kann durch deutliche Verringerung des fibrosierten Fettgewebes als Vorbereitung für eine physiologische Rekonstruktion mittels freiem Lymphknotentransfer eingesetzt werden, oder im Stadium III zur Gewichtsentlastung der betroffenen Extremität und leichteren Kompressionsbestrumpfung verhelfen.

#### Direkte Exzision

Die Lymphchirurgie arbeitet mit bis zu 50-facher Vergrößerungen

Das direkte Entfernen betroffenen Gewebes, auch Charles-Verfahren genannt, wurde erstmals 1912 beschrieben. Es beschreibt die radikale, zirkuläre epifasziale Exzision von Haut und Unterhautgewebe gefolgt von einer Hautransplantation. Weitere Methoden des direkten Entfernens, sind das Sistrunk-Verfahren, welches Haut und Unterhaut entfernt, jedoch einen Direktverschluss an der medialen Extremitätenseite ermöglicht, sowie die Thompson-Operation, welche einen lateralen Zugang verwendet und ebenso direkt verschlossen wird. Diese Techniken sind äußerst schwerwiegenden Fällen des Lymphödems vorbehalten und finden heutzutage kaum Anwendung [2]. Trotz erfolgreicher Reduktion des Lymphödems gehen diese Techniken mit schlechten ästhetischen Ergebnissen einher.

#### Liposuktion

Die Liposuktion, oder Fettabsaugung, dient der Volumsverringerung der betroffenen Extremität und stellt keine spezifische Therapie in der Lymphödemchirurgie dar. Jedoch kann bei bereits stattgefundenen Umwandlungen des Unterhautgewebes, die Liposuktion die Veränderungen reduzieren. Nach einer Liposuktion müssen Patienten permanent eine Kompressionswäsche tragen, welche das gesamte operierte Gebiet abdeckt, um einer weiteren Flüssigkeitsakkumulation entgegenzuwirken. Ein Fortführen von Lymphdrainagen ist ab ca. 4 Wochen postoperativ wieder möglich.

#### Lymphovenöse Anastomose

Eine Form der physiologischen Rekonstruktion bei Lymphödemen, stellt die Anastomose (chirurgische Verbindung) von Lymphgefäßen an Venen dar. Durch diese Verbindung kann die Lymphe frühzeitig und schneller in das venöse System abfließen [16]. Die Hauptindikation zur Anlage einer lymphovenösen Anastomose (LVA), ist das obstruktive Lymphödem.

Durch die Entwicklung der modernen Mikrochirurgie sind radikale Therapien in den Hintergrund gerückt

Zur präoperativen Planung der Anastomosenstellen wird ICG subkutan appliziert. Durch die Darstellung mittels Infrarotkamera werden ungestörte Lymphgefäße als klare Linien sowie Stellen mit gestörtem Abfluss, als Wolke oder Stern (wegen eines Rückflusses über die Haut) dargestellt (Abb. 2). Passagen mit klar definierten Unterbrechungen entsprechen der Kreuzung von Lymphgefäßen mit Venen, welche ICG nicht aufnehmen und somit nicht aufleuchten. Diese Punkte dienen als zukünftige Anastomosestellen.

Zur intraoperativen Darstellung des Lymphabflusses kann erneut ICG injiziert werden. Sowohl das primäre als auch das sekundäre Lymphödem profitieren von diesem Bypasseingriff.

VLNT’s und mikrovaskuläre Lappenplastiken können, im Zuge von Brustrekonstruktionen, kombiniert werden

**Abb. 3:**
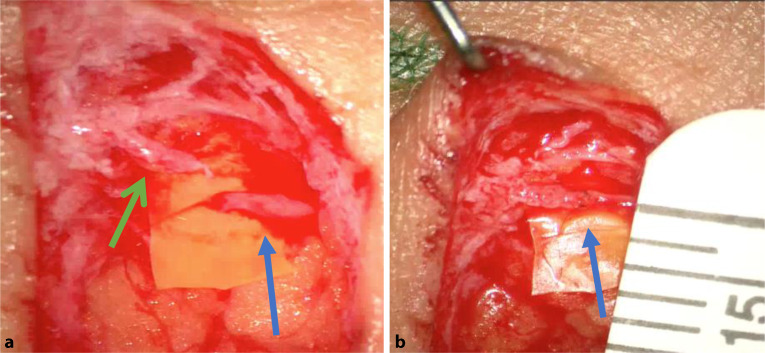
**a** Präparation des Lymphgefäßes (*grüner Pfeil*, Größe ca. 0,5 mm) und der Vene (*blauer Pfeil*, Größe ca. 0,5 mm), **b** Anlage der Lymphovenösen End-zu-End Anastomose (*blauer Pfeil*). Lineal zum Größenvergleich

Zu Beginn des Eingriffes wird an den vorher mit ICG bestimmten Anastomosenstelle ein ca. 2–3 cm langer, querverlaufender Schnitt gesetzt. Das präoperativ angefärbte Lymphgefäß wird aufgesucht, mit Mikroinstrumentarium freipräpariert und abgesetzt. Anschließend wird eine kaliberstarke Hautvene präpariert, abgesetzt (Abb. 3a) und mit einer Fadenstärke von 11‑0 (kleiner als das menschlich Haar), End-zu-End oder End-zu-Seit anastomosiert (Abb. 3b) [17, 18]. Eine Überprüfung der Dichtigkeit und des erwünschten ungestörten Abflusses, wird erneut mit ICG überprüft.

#### Vaskularisierter Lymphknotentransfer (= freie Lappenplastik)

Diese Operationstechnik kommt ab Stadium II oder höher zum Einsatz, wenn eine totale Okklusion des Lymphsystems, eine partielle Okklusion mit frustraner konservativer Therapie, oder rezidivierende Cellulitiden vorliegen [19].

Die Liposuktion dient allem voran der Volumenreduktion

**Abb. 4:**
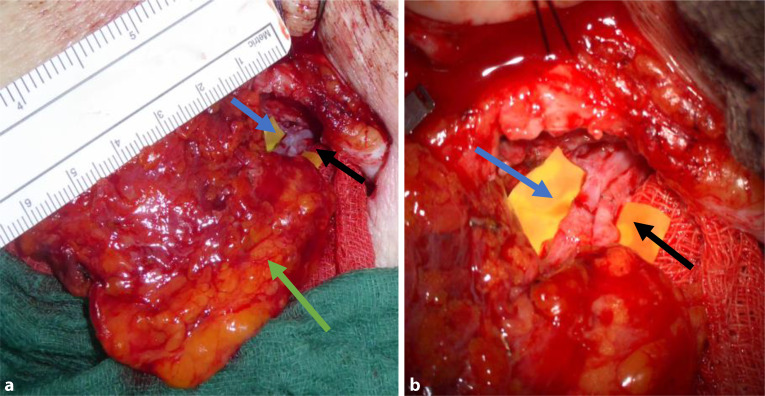
**a** Vaskularisierter Lymphknotenlappen (*grüner Pfeil*) vor dem Einnähen ins Empfängerbett. Im Hintergrund sind die arterielle (*schwarzer Pfeil*) und venöse (*blauer Pfeil*) Anastomose sichtbar, **b** Arterieller (*schwarzer Pfeil*) und venöser (*blauer Pfeil*) Anschluss des VLNT

Freie mikrochirurgisch transferierte Lymphknotentransfers sind Lymphknotenpakete, welche in umliegendem Fettgewebe eingebettet, als Lymphknotenlappen transplantiert werden. Dabei werden nur Arterien und Venen vernäht, nicht jedoch Lymphgefäße (Abb. 4). Diese führen am Transplantationsort zu einer erhöhten Drainage der umliegenden interstitiellen Flüssigkeit, welche sie dem angeschlossenem Venensystem zuführen [20]. Transferierte Lymphknoten wirken somit als Pumpe und unterstützen den Abtransport von Lymphflüssigkeit aus dem umliegende Gewebe.

**Abb. 5:**
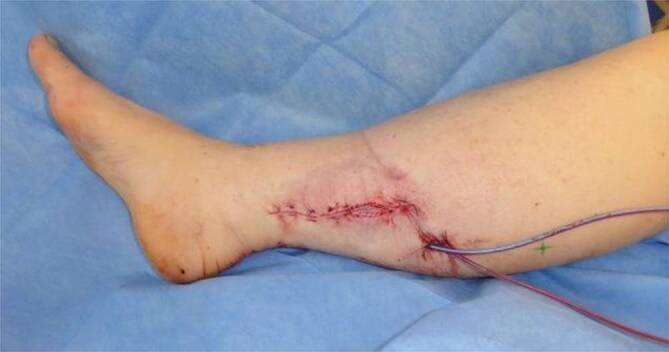
Eingebetteter Lymphknotenlappen aus der Axilla. Entlastung des Lappens durch Debulking an der Empfängerstelle (Entfernung von umliegendem Gewebe) und Deckung mittels Spalthauttransplantation. Das Anschneiden von Lymphgefäßen im Zuge des Debulkings regt zusätzlich die Lymphangiogenese an

Je nach Empfängerstelle kann es notwendig sein den Hautmantel des Lymphknotenlappens zur Integumenterweiterung mit zu transferieren. Dies ist bspw. am Handgelenk, oder Knöchel notwendig. An Lokalisationen mit mehr Weichteilmantel kann der Lymphknotenlappen entweder vollständig eingebracht oder auch mit Spalthaut bedeckt werden (Abb. 5).

Eine präoperative subkutane Injektion von ICG hilft bei der Darstellung von Lymphgefäßen

An den Empfängerstellen produzieren freie Lymphknotenlappen VEGF, welches zur Lymphangiogenese und folglich zur Verbindung von lymphatischen Bahnen mit dem Lymphknotenlappen führt. Ebenso erzielt eine Resektion von Narbengewebe an der Empfängerstelle eine Entlastung des Lymphknotenlappens. Diese Entlastung möglicher Obstruktionen, legt Lymphgefäße frei und fördert zusätzlich die Lymphangiogenese.

Transferierte Lymphknoten saugen, ähnlich wie ein Schwamm, umliegende Flüssigkeit auf

Bei der Transplantation von freien Lymphknotenpaketen ist postoperativ ein engmaschiges Monitoring indiziert, um die Durchblutung bis zum sicheren Einheilen zu überwachen. In den ersten postoperativen Tagen ist eine eingeschränkte Bettruhe erforderlich und die entsprechende Extremität sollte hoch, bzw. auf Herzhöhe gelagert werden.

## Postoperatives Management

Lymphknotenlappen sollen nur dann entnommen werden, wenn daraus kein Lymphödem der Hebestelle resultiert

ICG wird zur Überprüfung der Anastomosendurchgängigkeit verwendet

**Abb. 6:**
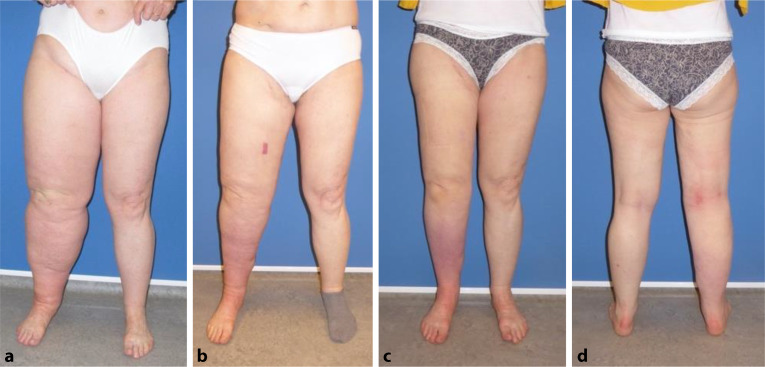
Lymphödem nach Uteruskarzinom mit Lymphknotendissektion der rechten unteren Extremität bei einer 62-jährigen Frau (Abb. 1b und 5). **a** Ausgangsbefund. **b** 2 Monate nach freiem Lymphknotentransfer. Die Spalthautentnahmestelle ist als livides Rechteck erkennbar. Man bemerke, der Lymphknotenlappen aus Abb. 5 ist kaum zu sehen. **c** und **d** 12 Monate postoperativ. Die Patientin trug kontinuierlich Kompressionswäsche der Klasse II Flachstrick nach Maß

Eine manuelle Lymphdrainage sollte, wenn möglich täglich, ab dem ersten postoperativen Tag im stationären Bereich erfolgen. Nach der Entlassung sollte eine Lymphdrainage alle zwei Tage für die ersten 6 Wochen durchgeführt werden. Danach muss diese für weitere 3 Monate zweimal wöchentlich erfolgen. Postoperativ sollte eine mäßige Kompression der entsprechenden Extremität mit Kurzzugbandagen, mit nicht zu starkem Zug außerhalb der KPE, etabliert und Patienten sollten nach der Entlassung mit Flachstrick Kompressionswäsche nach Maß der Klasse II versorgt werden. Zwölf Monate nach dem Eingriff kann ein sukzessives Entwöhnen der Kompressionswäsche erfolgen. Regelmäßige ambulante Kontrollen sind initial zur Überprüfung des Fortschrittes notwendig (Abb. 6).

Ein Kuraufenthalt in einer auf die Behandlung von Lymphödemen spezialisierten Rehabilitationsklinik ist zu empfehlen.

## Conclusio

Das Lymphödem stellt nicht selten eine Folge nach onkologischen Therapien im Kopf-Halsbereich, gynäkologischen Bereich (Brust, Ovarien, Uterus) aber auch nach Prostatakarzinomen dar. Vor allem, onkologische Eingriffe am Lymphsystem in Kombination mit Strahlentherapie können bei einer signifikanten Anzahl an Patienten zu Lymphödemen führen. Durch moderne Ansätze in der Diagnostik kann das Lymphödem frühzeitig erkannt und als Basistherapie konservativ durch eine KPE oft verbessert werden. Ab Stadium I sollte jedoch die Vorstellung an einer, auf die Behandlung von Lymphödemen spezialisierten Plastisch-Rekonstruktiven Abteilung erfolgen, um eventuell notwendige physiologische mikrochirurgische Rekonstruktionen rechtzeitig in die umfassende therapeutischen Versorgung der Patienten einzuplanen. Diese führen bei meist kurzen stationären Aufenthalten und hoher Erfolgsrate zur deutlichen Besserung der Beschwerdesymptomatik.

Eine frühzeitige Absprache mit den zusammenarbeitenden Abteilungen (z. B.: Physikalische Medizin, Nuklearmedizin) und eine zeitgerechte Planung der postoperativen Therapiemaßnahmen sind wichtige Voraussetzungen für eine optimierte interdisziplinäre Behandlung der Patienten.

## DFP-Fragen

Was ist die häufigste Ursache des sekundären Lymphödems in Entwicklungsländern?

Brustkrebs

Filariose

Genetische Mutationen

Traumata

Alle der oben genannten

Wie hoch ist die Inzidenz sekundärer Lymphödeme bei Brustkrebs?

7 %

10,5 %

15,2 %

19,9 %

20 %

Welche Genmutation liegt meist bei primären Lymphödemen vor?

VEGF

SOX18

PDGF

NGF

EGF

Warum resultieren bei Druck auf die Haut im Stadium II des Lymphödems keine Hautdellen?

Wegen Apoptose von Lymphgefäßen

Als Resultat der KPE

Bedingt durch Liposuktion

In jedem Lymphstadium verbleibt eine Hautdelle auf Druck

Aufgrund des fibrotischen Umbaus im Unterhautgewebe

Wie viele Grundgriffe beinhaltet die manuelle Lymphdrainage nach Vodder?

1

2

3

4

5

Welches radioaktive Isotop wird im Zuge der Lymphszintigraphie verwendet?

Technetium-98

Phoshpor-32

Technetium-99

Iod-131

ICG

Was ist die Basis der Lymphtherapie?

LVA

Direkte Exzision

Liposuktion

VLNT

KPE

Welche dieser Techniken wird nicht in der Lymphchirurgie verwendet?

Lymphvenöse Anastomosen

Freier vaskularisierter Lymphknotentransfer

Arterieninterponate

Radikale Exzision

Kombinierter mikrovaskulärer Brustaufbau mit freiem Lymphknotentransfer

Welche ist keiner der vier Grundgriffe der MLD nach Vodder?

stehender Kreis

Pumpgriff

Schöpfgriff

Drehgriff

Zuggriff

Welche Strukturen werden bei VLNT (vaskularisierter Lymphknotentransfer) nicht vernäht?

Arterien

Venen

Lymphgefäße

Haut

Keine der oben genannten
